# Physiotherapeutic Intervention Techniques for Knee Osteoarthritis: A Systematic Review

**DOI:** 10.7759/cureus.56817

**Published:** 2024-03-24

**Authors:** Kamya J Somaiya, Subrat Samal, Manali A Boob

**Affiliations:** 1 Musculoskeletal Physiotherapy, Ravi Nair Physiotherapy College, Datta Meghe Institute of Higher Education and Research, Wardha, IND

**Keywords:** current physiotherapy techniques, knee osteoarthritis, standard physiotherapy program, aquatic therapy, kinesiotaping

## Abstract

Globally, knee osteoarthritis (KOA) is the leading cause of disability. The most prevalent complaints associated with KOA are knee pain, joint stiffness, and weakness in the muscles of the lower limbs. These symptoms impede movement and result in functional limitations. As a result, people with KOA have a lower quality of life. For all patient groups with knee OA, an effective rehabilitation program focuses on improving knee range of motion, isometric quadriceps strength, and productivity level while reducing discomfort. The American College of Rheumatology (ACR) categorization criteria for KOA, research on KOA physiotherapy, and reviews covering various physical therapy interventions, including exercise, physical modalities, and patient education, were used to narrow down the pool of 180 systematic reviews to 15 articles. Google Scholar, PubMed, the Cochrane Library, and EMBASE were the databases that were used. The following keyword combinations were included in our search: KOA and physiotherapy or interventions or exercises, strengthening and stretching, concentric and eccentric training. Through our analysis, we identified a few methods that, in addition to standard therapy, could be used in clinical settings for people with osteoarthritis in the knee. It has been shown that Mulligan, Pilates, Kinesiotaping, Aquatic Therapy, and other current therapies are effective. The study employed a broad range of results. This review concludes that rather than relying solely on conventional therapy, it is preferable to combine a number of the most current physiotherapy techniques with it.

## Introduction and background

Osteoarthritis of the knee is the main reason for disability worldwide [1]. It is a chronic deteriorating condition that affects the knee joint. It is signified by anatomical and physiological abnormalities that cause bone tissue to change position, osteophytes to form, synovial membrane inflammation, ligament damage, and loss of normal joint movement [2]. People with KOA experience a reduction in quality of life as a result [1]. The most common KOA-related complaints are knee pain, joint stiffness, and lower limb muscle weakness, all of which limit movements and cause functional restrictions [1]. As people live longer around the world, there is an increase in the prevalence of knee osteoarthritis (KOA) [3]. The combined worldwide prevalence of KOA was 22.9% in those over 40 and 16.0% in those over 15 years of age. Accordingly, the global population with KOA in 2020 will be around 654.1 million people (40 years of age and older). The combined worldwide incidence of KOA in those 20 years of age and older was 203 per 10,000 person-years (95% CI, 106,331) [4]. By 2050, the number of cases of osteoarthritis (OA) is expected to rise from 2020 to 74.9% (59.4-89.9) for the knee, 48.6% (35.9-67.1) for the hand, 78.6% (57.7-105.3) for the hip, and 95.1% (68.1-135.0) for other types [5]. The literature indicates that women experience KOA more frequently. In these investigations, the overall proportion of KOA varied from 27.1% to 66.1%, depending on the lower age limit of the study group [3]. KOA has a number of reasons. The following categories apply to knee osteoarthritis based on their causes such as idiopathic, which can be both localized and generalized KOA, or secondary OA, which can result from diseases such as calcium deposition, congenital or developmental disorders, post-traumatic OA, or developmental disorders [6].

Pathophysiology of KOA

The diarthrodial joint unites two adjacent bones and is protected by a synovial bursa and a unique layer of articular cartilage [7]. More than half of undetectable people over the age of 50 have subchondral bone marrow lesions (SBMLs), which are caused by aberrant and chronic mechanical assaults and result in cellular and biomolecular reactions to microfractures, reported by abnormal MRI signals below the calcified cartilage [8].

Matrix metalloproteinase (MMP) synthesis is diminished by macrophage reduction and neutralization of macrophage-derived TNF and IL-1, which associate synovial macrophages with cartilage deterioration [9]. Transforming Growth Factor (TGF), Bone Morphogenetic Proteins (BMP-2), and BMP-4, macrophage-derived growth factors that are involved in chondrogenesis and bone formation and are indicative of wound recovery responses, were found to have a negative impact on pathologic bone formation in OA [9]. This indicates that the impact of inflammation in OA is multifaceted and regulated by the triggering stimuli. Still, it also influences macrophage-mediated inflammatory activity in the pathologic cartilage and bone responses associated with OA [9].

It has been established that the development of OA includes the wear and tear of the cartilage extracellular matrix (ECM), which, along with bone remodeling, causes progressive degradation of the joints and structural failure [10]. The conclusion that MMPs mediate the breaking down of type II collagen and that aggrecan is destroyed by related metalloproteinases called adamalysins with thrombospondin motifs (ADAMTSs) is supported by a substantial body of evidence [10].

Clinical features of KOA

KOA is characterized by apprehension decreased range of motion (ROM), stiffness and edema of the joint, weak muscles, and joint instability [11]. Joint discomfort that worsens with movement and is reduced by rest is one of the typical signs of OA, as is momentary joint stiffness that appears after inactivity. Physical evaluations of people with OA may reveal “knobby” joints as a result of the reorganization of bone and cartilage [12]. According to the latest research, knee crepitus is a quick and reliable test that predicts the progression of symptomatic KOA over time.

Diagnostic investigations

Rather than clinical characteristics, radiographic appearance is frequently used to make the diagnosis of KOA. In 1957, Kellgren and Lawrence advocated the use of radiographic appearance [6]. Five grades (0, normal to 4, severe) were used by Kellgren and Lawrence to categorize OA. According to this scale, grade 1 demonstrates that there are no bony growths and normal joint space; grade 2 demonstrates that there may be a slight narrowing of the joint space and the emergence of bony growths; grade 3 demonstrates that there is a narrowing of the joint space and mild bony growth formation; and grade 4 demonstrates that there is moderate bony growth formation, a narrowing of the joint space, and sclerosis of the subchondral bone [13].

Physical therapy management

All KOA groups respond well to a condition-specific rehabilitation program that reduces discomfort severity while enhancing knee range of motion, isometric quadriceps strength, and level of functional efficiency [14]. In those with KOA, systematic quadriceps isometric contraction exercise successfully reduced pain and enhanced knee joint function [15]. Interferential therapy with the specified parameters is proved to be very effective in improving pain [16]. People with KOA may find stretching procedures helpful for managing their pain, especially when done on their own [17]. In this way, numerous studies have demonstrated the significance of conventional physical therapy rehabilitation programs for individuals with KOA.

People with KOA have been established to greatly advantage from a variety of modern physiotherapy intervention options. Aquatic therapies, mulligan mobilization, pilates, closed-chain exercises, task-specific perturbation training, etc., are a few techniques. These strategies, as well as others that have been demonstrated to be extremely useful in such groups, are supported by a wealth of evidence. In this review, we have studied the many novel methods that, when used in conjunction with standard treatment, can benefit people with KOA.

## Review

The approach of searching strategy through electronic databases for English language literature studies in which different physiotherapeutic regimens were used in individuals with KAO. The databases utilized were Google Scholar, PubMed, Cochrane Library, and EMBASE. We searched for the following combinations of keywords: KAO and physiotherapy or interventions or exercises, concentric and eccentric training, stretching, and strengthening. Numerous physiotherapeutic studies on KAO were included in the searches, but randomized control trials (RCTs), taking the year of publication into account, received more attention. Out of 180 systematic reviews, 15 articles were selected from the timeframe year 2019 to July 2023 based on the eligibility criteria as follows.

Eligibility criteria

The articles that needed to be examined were chosen based on the inclusion and exclusion criteria stated underneath. The inclusion criteria were as follows: studies included participants who met the American College of Rheumatology (ACR) categorization criteria for KAO, research on KAO physiotherapy, the reviews that covered various varieties of physical therapy interventions, such as exercise, physical modalities, and patient education, the key outcomes for this overview are pain and physical function, but we have also included psychological outcomes (such as scales of psychological disability or self-efficacy) because patients may find this information to be significant. The exclusion criteria were as follows: studies conducted that were not available in English; studies conducted without using human participants; studies for which there was no full text accessible; studies whose objectives were not related to the review; studies whose interventions were not clearly explained; studies based on the medications and studies whose outcome measures used were not reliable.

Data extraction and outcome measures

Two reviewers extracted data from the chosen publications. The first author, year of publication, outcome measures used in the study, the population included in the study, type of the study, i.e., randomized control trials, experimental studies, comparative studies, and prospective studies, specifics regarding the interventions administered, results of the study, conclusion of the study, and an analysis by the reviewer regarding the article were the data that were extracted from the selected articles. The research employed a variety of outcomes. The visual analog scale (VAS), the Western Ontario and McMaster Universities (WOMAC) pain subscales, the WOMAC physical function score, the Lequesne index (LI), the active range of motion (ROM), and the 50-meter walking time were the few outcomes. Assessment of these outcome based on the pre and post-intervention score. Selected studies are compared and summarized on the basis of the author's experience, existing theories, and models.

Result

**Figure 1:**
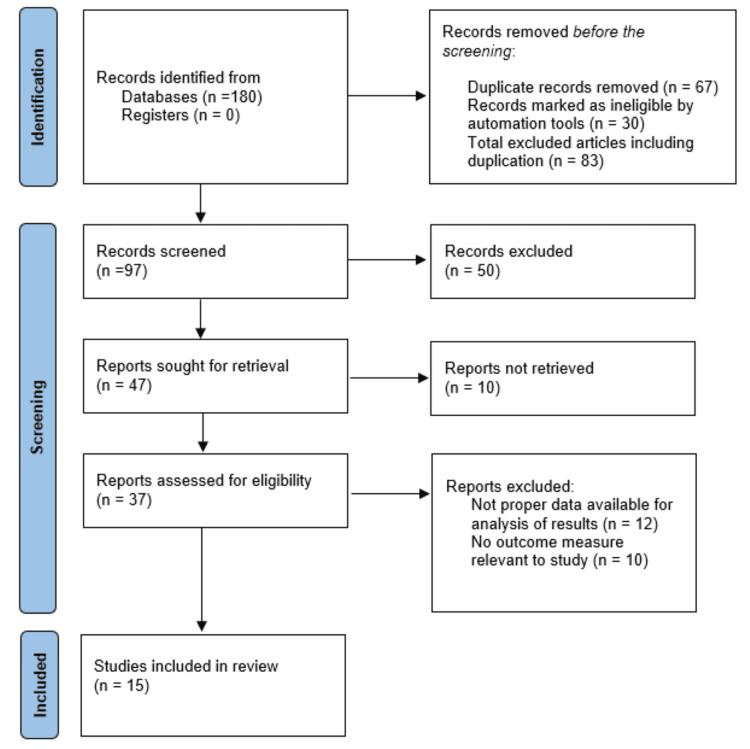
Prisma chart

Two reviewers evaluated which articles to include. They were identified in accordance with the study's title and abstract. A total 180 papers were shortlisted based on the study's title and abstract. Each of the two reviewers read the papers in its entirety. We sorted 15 articles based on the inclusion and exclusion criteria. The literature matrix was then updated to add these 15 publications. The review follows the Prisma guidelines. Figure 1 depicts the PRISMA flow diagram. Table 1 includes a reference to the literature matrix. This study provided compelling evidence for a number of novel intervention approaches, including kinesiotaping, non-thrust manipulation to reduce pain, muscle energy technique to increase knee extension at the end range, blood flow restriction therapy, eccentric exercises, pilates, aquatic therapy, which changes muscle strength and improves balance, and sensor-based gait training, which is a very useful tool for providing patients with virtual feedback and would be especially helpful for those with OA in the knee. However, these approaches can be used to enhance these programs and raise the standard of care they provide.

**Table 1:** Literature matrix MET - muscle energy technique, TENS - transcutaneous electrical nerve stimulation, KOA - knee osteoarthritis, VAS - visual analogues scale, WOMAC - western ontario and mcmaster universities arthritis index, ROM - range of motion, ACSM - american collage of sports medicine, PKE - passive knee extension test, PMAX - maximum power MMAX- maximum moment , ESWT - extracorporeal shockwave therapy, PNF - proprioceptive neuromuscular facilitation, MMP - matrix metalloproteinases, COMP - cartilage oligomeric matrix protein, KAM- Knee Adduction Moment , VASp- visual analogue pain scale, KOOS - knee injury and osteoarthritis outcome score, ICRT - isotonic concentric resistance training, IERT - isotonic eccentric resistance training, TRX - total resistance exercises, TUG - timed up and go, KI-knee instability , RM - repetition maximum, MIRT - moderate intensity resistance training, BFR - blood flow restriction therapy, OA - osteoarthritis, BBS - biodex balance system.

Sr. No	Author	Study Type	Study Sample	Intervention	Results	Conclusion	Analysis
1.	Anam et al. 2023 [18]	Comparative Study	The study incorporated 30 participants who met the inclusion requirements.	Kinesiotaping was utilised in conjunction with traditional therapy in Group A. Group B obtained Muscle Energy Technique along with standard treatment.	The study found that Group A improved substantially more than Group B in terms of hamstring flexibility and knee discomfort. Therefore, in patients with OA knees, the intervention in Group A was more efficacious in reducing pain and increasing knee extension range of motion and hamstring flexibility.	When compared to Group B (Muscle energy technique with conventional therapy), Group A (Kinesio taping with conventional therapy) had a substantial improvement.	Patients who have osteoarthritis of the knee receive advantages from conventional physiotherapy. However, in such instances, combining kinesiotaping with the standard treatment is more beneficial
2.	Witwit et al. 2022 [19]	A Randomized Clinical Trial	There were 42 patients exhibiting grade II primary knee osteoarthritis.	Non-thrust manipulation techniques were implemented on Group A. Group B was administered MET. The identical guided workouts and TENS program were provided to both groups.	The analytical findings of the comparisons between the groups revealed that there were major variations in the means of improvement of the VAS at rest and during exercise, WOMAC scores, PKE ROM, 30CST, and TFA. There wasn't much of a difference, other from the 6MWT distance.	MET and non-thrust manipulation are both efficient management techniques for KOA. Non-thrust manipulation may be implemented to improve pain and physical function with greater efficacy. Patients with KOA could gain more from MET in terms of improved knee extension range of motion.	Non-thrust manipulation technique is more successful in treating such conditions when focusing on reducing discomfort and physical complaints. Muscle Energy Technique is highly helpful while concentrating on knee extension range of motion.
3.	Trojani et al. 2022 [20]	Randomized Prospective Study	The study encompasses 60 participants.	With the exception of the muscle-strengthening activities (concentric versus eccentric), the exercise sessions for the two groups were analogous according to ACSM. Resistance bands and weights were employed by both groups to put greater stresses on the muscles.	In the WOMAC, both groups demonstrated substantial gains without any differences between them. Only the eccentric group showed a discernible improvement in the change in PMAX and MMAX at high velocity. Only the eccentric group had a quantifiable vastus medialis hypertrophy.	Patients with symptomatic knee osteoarthritis may reap advantages from eccentric or concentric training in terms of function and pain. More specifically, eccentric therapy alone has been demonstrated for enhancing vastus medialis volume and muscle performance.	Both eccentric and concentric exercise serve a purpose for developing strength, however eccentric exercise is preferred and ought to be used particularly while focusing on the vastus medialis.
4.	Ko et al. (2022 ) [21]	Randomized Controlled Study	Bilateral knee OA patients were enlisted in the study.	The Duolith SD1 device was utilised to deliver shockwave treatments. Weekly intervals separated the three shockwave treatment sessions for each subject. Direct application of the shockwave probe was made to the knee joint's patellofemoral border and medial tibial plateau's most sensitive spots. The same therapy was given to both knees.	The VAS score had declined substantially after 4 weeks in both groups, although the f-ESWT group had experienced a higher decline. The secondary outcomes in both groups enhanced substantially however the VAS score, WOMAC score, and 6-minute walk test enhanced more in the f-ESWT group. Our findings suggested that f-ESWT was superior than r-ESWT in treating knee osteoarthritis patients' pain and physical function.	When compared to the r-ESWT, the f-ESWT produced a greater reduction in pain as well as enhancements in physical function and the 6-minute walk test distance.	Patients with knee OA may receive shockwave therapy. Such patients can greatly advantage from focused extracorporeal therapy in terms of reducing pain and restoring physical function.
5.	Garbi et.al 2021 [22]	Prospective, Quantitative and Analytical Clinical trial of Randomized Control	29 patients who met the ACR criteria for OA and were 60 years of age or older	The following was provided as the intervention group protocol: Heating Muscle Strengthening for Quadriceps and Hamstrings Stretching for Buttock , Quadriceps , Ischiotibialis and Sural triceps. The Control Group did not procure any physiotherapy for two months.	In respect to the Control Group, subjects in the Intervention group showed statistically substantial improvements in pain, stiffness, and physical impairment as well as mobility characteristics.	When compared to senior people with knee OA who did not get any physiotherapy treatment, participants in the AP programme showed substantial improvements in pain, stiffness, functional capacity, gait time, and mobility.	Aquatic treatment is highly beneficial in reducing pain, improving joint mobility, and promoting an enhancement in quality of life.
6.	Meenakshi et.al 2021 [23]	Prospective study	68 participants with osteoarthritis of the knee in grades 1 and 2 participated.	Group 1 was provided Pilates drills Group 2 was provided Exercises with a Closed Kinematic Chain	When comparing pre-test to post-test data for participants with knee osteoarthritis, (Group-I) Pilates exercises and (Group-II) Closed Kinematic Chain exercises demonstrated statistically substantial distinctions within the groups.	According to the findings of the current study, in contrast to closed kinematic chain exercises, six weeks of Pilates exercise interventions considerably enhanced pain reduction, muscle strength, and functional performance.	In individuals with knee OA, Pilates exercises have been determined to considerably enhance pain, muscle strength, and functional performance.
7.	Masekar et al. 2021 [24]	Experimental Study	Using the inclusion criteria, 36 patients were identified.	Group A: PNF stretching Hold Relax (2 sets of 5 repetitions) using the proprioceptive neuromuscular facilitation approach Group B: Muscle energy technique, which incorporates post-isometric relaxation and reciprocal inhibition for two sets of five repetitions each.	PNF-treated Group A significantly outperformed Group B on the NPRS, Active Knee Extension Test, and WOMAC.	Both PNF stretching and MET are successful, however PNF stretching was substantially more successful in alleviating pain, enhancing hamstring muscle flexibility, and boosting functional mobility in comparison to MET.	PNF stretching was incorporated into the protocol for treating OA knee patients, and the results were more appealing in terms of hamstring flexibility improvement, pain relief, and independent functional mobility.
8.	Oguz et al. 2021 [25]	Randomized, Double Centered Study	Osteoarthritis of the knee has been detected in 22 female cases.	Six weeks of three days a week of training constituted the exercise programme. Over the course of the study's six weeks, the same skilled physiotherapist executed kinesiotaping three times per week.	Following the therapies, pain and functioning levels drastically rose in both groups. When COMP, MMP-1, and MMP-3 levels were assessed to rest in both groups prior to and after the intervention, they were greater after walking activity.	The levels of COMP, MMP-1, and MMP-3 did not alter, but exercise training and exercise training combined with kinesiotaping improved pain and physical function.	Both kinesiotaping and exercise training are very efficient at enhancing physical function and reducing pain. However, combining the two therapies yields better results.
9.	Wang et al. 2021 [26]	Prospective, Participant-Blinded, Parallel-Group Randomized Controlled Trial	71 individuals with grade I or II Kellgren-Lawrence early medial knee OA were enlisted.	Gait Retraining Group consisted of a one-session per week, six-week long gait retraining programme for KAM modulation. The walking exercise group's participants had to walk at their own tempo on an identical treadmill while wearing the identical pair of shoes.	KAM1 and VASP substantially dropped after gait retraining, whereas KOOS improved substantially, whereas these parameters remained the same in the walking exercise group. KAM2, KAAI, and KFM did not alter over time in either group.	In individuals suffering from early medial knee OA, the sensor-based gait retraining with real-time visual KAM curve feedback had an instantaneous impact on lowering KAM1 and KAAI, reducing knee discomfort, and enhancing functioning. Our research results offered a proof-of-concept for improving the existing clinical approach to treating patients with early medial knee OA without the need for expensive laboratory apparatus.	An essential component of the rehabilitation programme is gait training. A cutting-edge technology that we could use in practise to assist patients with knee osteoarthritis is a sensor-based gait retraining program.
10.	Afzal et al. 2021 [27]	Randomized Clinical Trial	A total of 40 obese female KOA (grade 2 and 3) patients who were between the ages of 40 and 60 were selected.	For eight weeks, the Isotonic Eccentric Resistance Training (IERT) group and the Isotonic Concentric Resistance Training (ICRT) group undertook IERT exercises and ICRT, respectively.	When compared to ICRT, IERT showed more advancement.	Both the IERT and ICRT exercise programmes possess advantages for strengthening the quadriceps muscles, although in individuals with knee osteoarthritis, the IERT programme is far more efficient than the ICRT programme.	IERT was far more effective than the other two types of resistance training in alleviating pain and stiffness by enhancing muscle strength, range of motion, and functional daily living activities. This study emphasises the need of including both activities in the therapy of KOA.
11.	Assar et al. 2020 [28]	Single Blinded, Randomized Control trial	36 patients suffering from a Kellgren-Lawrence Grade II radiographic grading of KOA were considered.	For the exercises in Total Resistance Training, the TRX® Rip Trainer TM(China) was the device of choice. For eight weeks, three times per week, there were a total of 24 sessions with each session lasting 60 minutes. A total of 24 sessions totalling eight weeks of aquatic exercise intervention were accomplished, each lasting exactly 90 minutes.	The results indicated that KI, VAS, and BBS substantially rose with time in both the TRX and aquatic groups, but that only TRX significantly boosted WOMAC (stiffness), knee flexion ROM, and quadriceps strength over time. For the VAS, KI, and BBS, a post hoc test additionally indicated that there were substantial variations between treatments and control groups, while for WOMAC (stiffness), only TRX and control groups were found to vary significantly.	Exercises employing the TRX system had a greater impact on knee stiffness, quadriceps strength, and range of motion (ROM). As a result, physical therapists could suggest TRX intervention as a suitable regimen for the rehabilitation of KOA patients.	Patients with knee OA recover greatly from the TRX workouts in terms of pain and stiffness. It can be employed to this population and is potentially a useful new treatment for those with knee osteoarthritis.
12.	Jahanjoo et al. 2019 [29]	Single-Blinded Randomized Clinical Trial	60 individuals with primary knee osteoarthritis were enrolled.	Physical therapy was routinely provided to the physiotherapy (PT) group. Physical therapy as standard was integrated with balance training utilising the Biodex balance system (BBS) for the balance training group (BT).	The visual analogue scale (VAS) pain score, the Western Ontario and McMaster Universities Osteoarthritis (WOMAC) pain score, the WOMAC total score, the Lequesne index, and the Timed Up and Go (TUG) test score were all considerably different in the balance training group. The fall risk score produced similar results.	Patients with knee OA experienced higher pain alleviation and the development of functional capacities when balance training was combined with physical treatment.	Physical therapy is advantageous, however in patients with knee OA, it was more effective for pain and functional abilities when combined with balance training.
13.	Harper et al. 2019 [30]	Pilot Randomized Clinical trial	35 people of or over 60 years old with symptomatic knee osteoarthritis (OA) and physical constraints were recruited.	Following exercise recommendations for seniors with OA, the MIRT training group did four lower-extremity movements (leg press, leg extension, leg curl, and calf flexion) at 60% of 1RM. The precise same four lower-extremity movements were carried out by the low-load BFR resistance training group at 20% of 1RM with the addition of external compression on the proximal thigh of both legs.	Maximal isokinetic peak torque differed throughout three movement speeds, with a change from before to after training of 9.96 Nm and a mean difference across groups (BFR relative to MIRT) of 1.87 Nm. Though greater unpredictable reports of knee pain were recorded compared to BFR, most other directions preferred MIRT.	The results suggested that BFR could be used as a substitute exercise plan to alleviate pain and enhance older persons with knee OA. Despite the possibility that BFR is less effective than MIRT, a fully powered RCT is required to explicitly test this hypothesis.	BFR is a safe and practical substitute for elderly people with knee OA. However, further research is required to demonstrate their efficacy. Nevertheless, it has not been proven to be more advantageous than low intensity resistance exercise.
14.	Foucher et al. 2019 [31]	Prospective Cohort Study	Both 25 women without knee OA and 25 women with self-reported knee OA volunteered to take part in the study.	A microprocessor-controlled, stepper motor-driven dual-belt treadmill was employed in Trip-Specific Perturbation Training for delivering postural perturbations. Each participant became dynamically unstable in the forward direction due to the protocol's 22 postural perturbations, for which the belt accelerated posteriorly.	Trunk flexion angular velocity dropped by 193% in the OA group and by 32% in the control group, respectively, and enhanced from pre to post in the OA and control groups, respectively, exhibiting a shift to the direction of extension. The findings imply that trip-specific training can enhance women with knee OA's compensatory stepping response kinematics.	Women with knee OA demonstrated a substantial boost in their kinematics related to the recovery stepping response after just one session of trip-specific perturbation training.	Women with OA can benefit from a single trip-specific training session just as much as women in the control group in terms of improving the recovery kinematics necessary for a successful trip recovery, notably by regaining trunk control.
15.	Sedaghatnezhad et al. 2019 [32]	Randomized Clinical Trial	Participating were 30 patients with knee osteoarthritis.	Physical therapy was delivered to the control and intervention groups over a 10-session period. The intervention group also received 30 minutes of 1.1 m/s walking on an 8-degree treadmill during each session. At baseline, post-treatment, and a 20-day follow-up, outcome variables such as pain, excursion ranges, stride length, and walking speed were assessed.	From baseline to post-treatment and from baseline to follow-up, only the intervention group's stride length and walking speed exhibited substantial modifications. Additionally, from baseline to follow-up, a substantial improvement in excursion ranges was observed solely in the intervention group.	This study showed that, in contrast to physical therapy alone, integrating uphill walking to physical therapy improves stride length and walking speed while also having long-lasting impacts on knee ranges, stride length, and walking speed.	It is preferable to integrate uphill treadmill training with traditional physiotherapy. Patients with knee osteoarthritis will have effective gait improvement.

Discussion

The systematic literature review focuses on a number of research analyses that assess the effects of different types of training programs on KAO. These study methods include randomised control trials, experimental studies, comparative studies, and prospective studies to assess features such as pain, range of motion, strength, physical function, and psychological consequences. We discovered that the variation in misalignment is influenced by additional parameters such as meniscal degeneration and position, bone attrition, osteophytes, and ligament injury [33]. Knee pain, its severity, and greater physical functional limits are all correlated cross-sectionally with poor proprioceptive acuity as measured by joint position awareness [34]. Based on the KL scoring criteria, there is systematic shift in the knee joint muscle recruitment sequence are changed during gait in a systematic manner as structural KOA severity increases. These changes in muscle activation patterns have been associated with systematic temporal response delays, higher demand for active stiffness throughout the gait cycle and particularly during mid-stance, and diminished medial compartment joint loading with increased structural severity [35].

The elderly with KOA who participated in the structured aquatic physiotherapy program demonstrated increases in functional capacity (FC) and mobility when compared to conventional physiotherapy. These findings are consistent with our review, which suggests that aquatic therapy is more effective in boosting and enhancing the individual's strength and performance capacity in daily life than conventional physiotherapy [22]. In comparison to Muscle Energy Technique with conventional therapy, Kinesio taping significantly improved pain on the VAS, increased range of motion on the Goniometer, and increased hamstring flexibility on the Active Knee Extension Test [18]. For adults with initial medial KOA, a sensor-based gait retraining program was more effective than walking exercises at reducing medial knee loading, relieving knee discomfort, and improving indicators of OA [26]. The kinematics associated with the recovery stepping response in women with KOA were dramatically enhanced by a single trip-specific perturbation training session [31]. Both pilates exercises and closed kinematic chain exercises significantly improved pain reduction, muscle strength, and functional performance after six weeks of intervention. As compared to the closed kinematic chain exercises the pilates approach boosts mechanoreceptor sensitivity, which amplifies reflex neuromuscular protection mechanisms. Pilates training has been tailored to improve general body coordination, increase skeletal muscle recruitment, encourage muscular co-contraction, and activate proprioception throughout the knee joint. These exercises precisely minimize pain and improve lower limb muscle strength, coordination, and adaptability, hence improving the individual's general well-being life. However, it was discovered that pilates exercises were more efficient than closed kinematic chain exercises [23].

This review suggests that people with KAO respond well to conventional physiotherapy rehabilitation programs. However, these programs can be improved, and their quality of care can be increased by using other strategies. Through this research, we gathered strong evidence for few new intervention strategies such as kinesiotaping, non-thrust manuplation to reduce pain, muscle energy technique to increase the end range knee extention, blood flow restriction therapy, eccentric exercises, pilates, aquatic therapy enchances the muscular strength which leads to improve balance and sensor based gait traning is very effective to give the virtual feedback to the patients this would be extremely beneficial for people with KAO. In order to improve patients' standard of living, this study gives a brief overview of therapies that can be used in addition to regular physiotherapy.

Study limitations

Only English-language articles that were available were included in this review. We sorted a rather small amount of reviews. It is necessary to do a larger investigation including many studies. Despite our best efforts, we were unable to incorporate all available papers from databases and periodicals. Small sample sizes and other methodological errors made it more difficult for the reviewer to manage the data. It was unable to rule out the possibility of selection bias in the review.

## Conclusions

In order to determine how various research affected individuals with KOA, we conducted an analysis of them based on our inclusion and exclusion criteria. We reviewed 15 of these studies that demonstrated various physiotherapy techniques that were successful with this population. Patients with KOA benefited greatly from these recent treatment methods. Kinesiotaping, aquatic therapy, mulligan, pilates, and other modern therapies are all documented to be successful. These methods, when used in conjunction with conventional physiotherapy, have shown to be quite effective in such patients. Through this review, we were able to highlight a few contemporary methods that could be used in conjunction with traditional treatment in a clinical setting to help people with KOA. This review comes to the conclusion that combining various modern physiotherapy techniques with conventional therapy is preferable to conventional therapy alone.
